# Triggering signaling pathways using F-actin self-organization

**DOI:** 10.1038/srep34657

**Published:** 2016-10-04

**Authors:** A. Colin, L. Bonnemay, C. Gayrard, J. Gautier, Z. Gueroui

**Affiliations:** 1 Ecole Normale Supérieure, Department of Chemistry PSL Research University-CNRS-ENS-UPMC 24, rue Lhomond, 75005, Paris, France

## Abstract

The spatiotemporal organization of proteins within cells is essential for cell fate behavior. Although it is known that the cytoskeleton is vital for numerous cellular functions, it remains unclear how cytoskeletal activity can shape and control signaling pathways in space and time throughout the cell cytoplasm. Here we show that F-actin self-organization can trigger signaling pathways by engineering two novel properties of the microfilament self-organization: (1) the confinement of signaling proteins and (2) their scaffolding along actin polymers. Using *in vitro* reconstitutions of cellular functions, we found that both the confinement of nanoparticle-based signaling platforms powered by F-actin contractility and the scaffolding of engineered signaling proteins along actin microfilaments can drive a signaling switch. Using Ran-dependent microtubule nucleation, we found that F-actin dynamics promotes the robust assembly of microtubules. Our *in vitro* assay is a first step towards the development of novel bottom-up strategies to decipher the interplay between cytoskeleton spatial organization and signaling pathway activity.

To achieve their numerous functionalities, cells have evolved a large variety of strategies to coordinate the spatial organization of intracellular components at multiple scales. The cytoplasm which is a highly crowded environment and heterogeneous at nearly all length scales participates in this spatial organization^1^. In particular, the specific physical properties of the cytoplasm have a strong impact on the spatiotemporal organization of signaling networks that are essential for cellular behavior^2^. Spatial heterogeneities in protein concentration can lead to the generation of gradients of enzymatic activities spanning over several micrometers and producing pockets of concentrated enzyme activity involved in cell morphogenesis^3^. In addition, the isolation of specific proteins into membrane-bound organelles, or their recruitment and immobilization into multiprotein complexes using scaffold proteins, allows the simultaneous binding of signaling proteins enhancing their interactions and promoting specific cellular functions^4^,^5^,^6^,^7^. For instance, recent studies have suggested how multivalency and cooperativity mediated by protein-protein or protein-RNA interactions could generate phase-separated micro-domains to isolate functional biomolecules into localized subcellular regions in the absence of membrane barriers and eventually triggering signaling activity^8^,^9^,^10^,^11^,^12^. Other studies describe P granules in *Caenorhabditis elegans* as liquid-like compartments and explain how biochemical reactions can be facilitated within the cytoplasm^13^. These studies provide an emerging picture on how phase transitions can be a driving force to organize the cell cytoplasm^9^.

The cytoskeleton can also organize signaling pathways in space and time by partitioning the cell and providing transient docking sites for proteins^14^,^15^. For example, actin microfilament dynamics regulate the clustering of membrane proteins controlling T cell signaling^16^,^17^ or the positioning of polarity markers during the establishment of the anterior-posterior axis in *C. elegans* embryos^18^. In a different context, subcompartmentalization of signaling proteins mediated by the actin cytoskeleton has been proposed to regulate dendritic spines during neuronal plasticity^19^. These recent works often describe mechanisms based on the interplay between F-actin operating at the membrane and signaling activity^14^. Although recent studies highlight novel roles of cytoplasmic F-actin in various cellular functions^20^,^21^,^22^,^23^,^24^,^25^,^26^, a general framework explaining how such cytoplasmic microfilaments may also coordinate signaling pathways in space and time in the cytoplasm is still missing.

*In vitro* reconstitutions of cellular processes have provided key breakthroughs for understanding the basic morphogenetic properties of cytoskeleton organizations^27^,^28^,^29^,^30^,^31^,^32^,^33^,^34^,^35^,^36^. These approaches also revealed how the physical and kinetic properties of the cytoskeletal elements determine their spatial self-organization^29^,^37^,^38^,^39^. In addition, studies deciphering the effect of the spatial boundaries on these assemblies shed the light on the role of geometric and mechanical parameters on fundamental organizing principles^36^,^40^,^41^,^42^,^43^,^44^,^45^. Here we examine how *in vitro* reconstitution of cellular processes can provide novel insights to decipher the interplay between cytoskeleton spatial organization and signaling pathway activity. In particular, we assess whether high-order organizations of cytoplasmic F-actin meshwork can trigger signaling activity by enhancing regulatory protein concentration. To test these hypotheses, we evaluated the enhancement of signaling activity: first as mediated by the membrane-free compartmentalization of nanoparticles operating as nanometric signaling platforms; and second, by the scaffolding of regulatory proteins by actin microfilaments (F-actin). We devised a bottom-up approach based on *Xenopus* egg extracts that have proven to be a powerful system to examine the self-organizing properties of the cytoskeleton^31^,^34^,^46^,^47^,^48^,^49^,^50^,^51^,^52^ as well as the spatiotemporal behavior of signaling cascades that regulate cell cycle events^53^,^54^,^55^,^56^,^57^.

Using metaphasic egg extracts confined within oil droplets, we can reconstitute the spontaneous generation of centripetal F-actin flow along with the formation of a contractile meshwork that eventually forms a ring-like organization (Fig. 1a). This F-actin flow drives the long-range transport of cytosolic components such as organelle-based structures as well as their partitioning within a confined space encompassed by filaments. To examine how cytoplasmic confinement could impact signaling activity, we first examined nanometric signaling platforms that are composed of regulatory proteins grafted to nanoparticles (Fig. 1b). As proof of concept we focused on Ran, a GTPase controlling the nucleation of microtubules, with an ultrasentitive switch activity characterized by a sigmoidal concentration dependency^58^,^59^,^60^,^61^ (Fig. 1c). RanGTP conjugated to selected nanoparticles was efficiently transported and concentrated by F-actin flow in confined egg extracts. Interestingly, we found that F-actin dynamics activate microtubule assembly after the local accumulation and the cytoplasmic partitioning of Ran-nanoparticles. This illustrates how F-actin self-organization can generate a robust biochemical switch by promoting localized fold increases in nanometric signaling platform concentration. We then evaluated the concept that F-actin operates as a polymeric hub to recruit regulatory proteins. We have engineered Ran to specifically recognize actin microfilaments using an actin binding protein based on utrophin. We found that the formation of a dense F-actin meshwork during contraction recruits Ran and triggers microtubule assembly. Altogether these studies shed the light on how cytoplasmic F-actin self-organization can promote a signaling switch based on concentration enhancement and polymeric scaffolding.

## Results

### Active confinement of cytoplasmic materials mediated by F-actin dynamics

**Figure 1:**
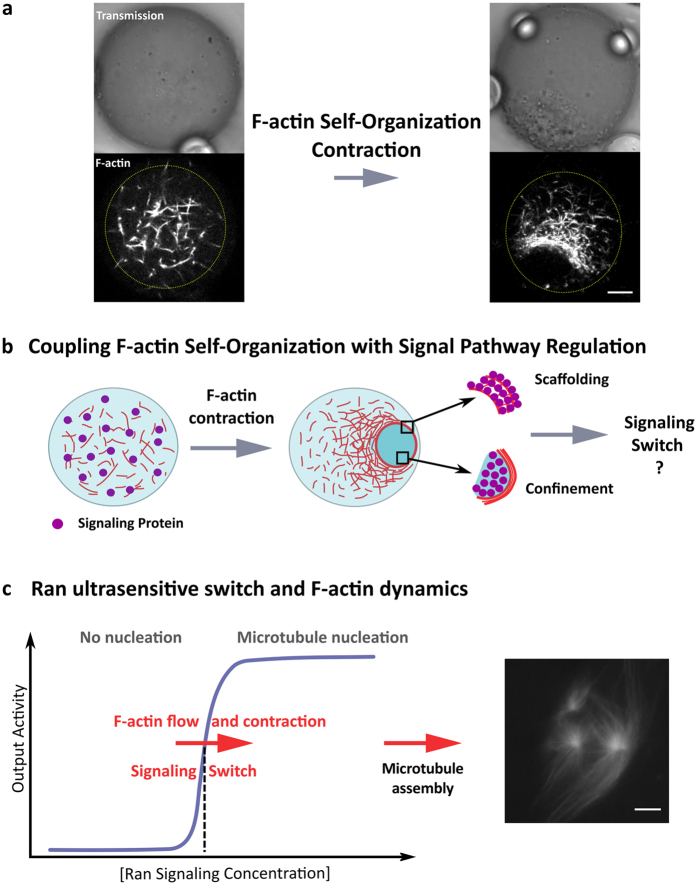
Membrane-free confinement mediated by F-actin self-organization to localize and trigger signaling pathways. (**a**) The confinement of egg extracts within oil droplets leads to the spontaneous generation of a centripetal F-actin flow that eventually forms a contractile ring-like structure. F-actin flow conveys cytoplasmic materials that are trapped by the filament meshwork. Actin microfilaments were labeled with Utr-GFP. Scale bar is 10 μm. (**b)** Schematic of the concept of signaling localization and switch: F-actin self-organization powered the active compartmentalization of nanoparticles operating as signaling platforms or the scaffolding of signaling proteins by F-actin polymers. Starting from a homogeneous distribution of signaling proteins, the generation of the F-actin flow drives the concentration enhancement of regulatory elements that could trigger signal pathway. (**c**) The activity of Ran GTPase is characterized by a non-linear response following a sigmoidal response with the concentration (ultrasensitive switch). The nucleation of microtubules is triggered above a concentration threshold of Ran. Our hypothesis is that the F-actin self-organization into a contractile meshwork will trigger the Ran signaling pathway and the nucleation of microtubules.

**Figure 2:**
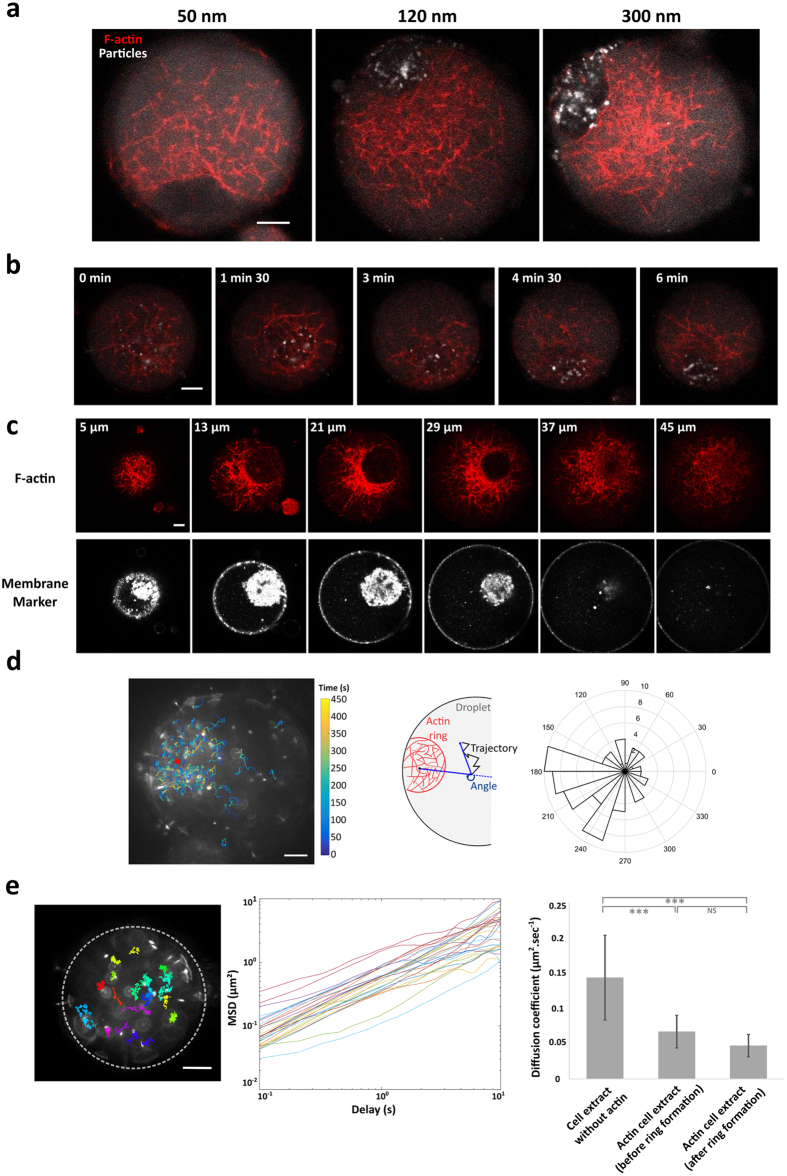
Characteristics of the membrane-free compartmentalization mediated by F-actin flow. (**a**) F-actin dynamics transport nanoparticles in a size-dependent manner. Accumulation of nanoparticles of different sizes (50 nm, 120 nm, and 300 nm) shows that only nanoparticles above a diameter of 100 nm are transported by F-actin flow. (**b**) Time-dependent accumulation of 120 nm fluorescent nanoparticles. After 6 minutes, almost all the nanoparticles are gathered at the center of the F-actin ring. (**c**) Confocal observations of F-actin ring-like structures show that microfilaments encompass cytoplasmic materials stained with membrane marker. Slices of a z-stack; images are 8 μm apart (from 5 to 45 μm in the droplet). (**d**) Left: Displacement field of 300 nm-nanoparticles reveals that their motions are highly directed towards the F-actin ring. Right: Angular distribution of tracked trajectories with respect to the actin ring position. (**e**) Estimation of the cytoplasmic diffusion coefficients in cell extracts incompetent for F-actin organization, and contractile cell extracts at early stage and late stage of F-actin ring organization. Mean and standard deviation are plotted. Scale bars are 10 μm.

To examine how cytoplasmic confinement and partitioning can be mediated by cytoskeleton, we investigated the organization of F-actin filaments into dynamic contractile patterns using Metaphase II *Xenopus* egg extracts encapsulated within emulsion droplets. To visualize the F-actin meshwork, we have supplemented *Xenopus* egg extracts with the calponin homology domain of utrophin fused to GFP (Utr-GFP) at sub-stoichiometric conditions (50 nM). Utrophin binds actin filaments with a reduced disturbance of the polymer assembly and dynamics^62^. In bulk extract (unconfined geometry), F-actin organized into a heterogeneous contractile meshwork within a few minutes (Supplementary Figure 1). In extracts confined into droplets, actin assembled into a convergent and centripetal contractile meshwork^41^. F-actin eventually formed a ring-like structure within 5 minutes with a dimension which scales to about one third of the droplet diameter for a broad size range (Fig. 1a, Supplementary Figure 2 and ref. 41). We estimated the size of the F-actin meshwork using confocal images of F-actin network (Supplementary Figure 3a). We found an average actin meshsize of about 1.1 μm and a range of interdistances between 0.5 to 4 μm (Supplementary Figure 3a). The F-actin meshsize decreases gradually from the periphery of the droplet to the actin ring and the mean F-actin contractile rate was about 90 nm.s^−1^ in agreement with previous results^41^ (Supplementary Figure 3b). The F-actin dynamics produced a long-range flow that convey micrometric objects such as microbeads over a distance covering several micrometers, in agreement with observations we previously reported^41^. In order to further characterize the system we examined the dynamics of the nanoparticle trapping. First, we analyzed the effect of particle size on the efficiency of accumulation (size ranging from 50 nm to 1 μm). Nanoparticles were covered with PEG-polymers to reduce non-specific interactions with cytoplasmic elements. We found that the majority of nanoparticles with a diameter larger than 100 nm can be trapped and efficiently conveyed by the F-actin flow (Fig. 2a). Small cytoplasmic proteins were not conveyed by F-actin flow, as confirmed by the absence of accumulation of fluorescent proteins within ring-like structures (Supplementary Figure 4). Our observations are reminiscent of cytoplasmic F-actin meshwork observed in starfish oocytes that produce a long-range transport of micrometer particles during chromosome congression^25^, suggesting a common mechanism of transport powered by cytoplasmic F-actin.

Time-lapse observations revealed that nanoparticle steric trapping occurs within 5 minutes (Fig. 2b and Movie 1). In addition, the use of a fluorescent membrane-tracker confirmed that organelle structures from the *Xenopus* cytoplasm accumulated in F-actin based-structures (Fig. 2c). To further characterize the confinement of nanoparticles, we monitored the spatiotemporal dynamic of individual nanoparticles during F-actin contraction (Supplementary Figure 5). Analysis of the mean orientation of the trajectories showed that the particle movements are directed towards the F-actin ring (Fig. 2d, Supplementary Figure 5a,b,c). The mean velocity of the transported particles is about 9.8 nm.s^−1^ (Supplementary Figure 5e), which is an order of magnitude smaller than the mean velocity of the F-actin flow (90 nm.s^−1^) (Supplementary Figure 3b). These results suggest that the trajectories of nanoparticles display a directed motion in intermittence with a Brownian-like behavior. Velocity fields along single nanoparticle trajectories are highly heterogeneous within droplets (Supplementary Figure 5d). For instance, the amplitude of the velocity along single tracked nanoparticles spans from approximately 20 nm.s^−1^ to approximately 150 nm.s^−1^ (Supplementary Figure 5f). In addition the distribution of instantaneous velocity is independent of the nanoparticle localization within the droplets (Supplementary Figure 5f).

Confocal observations showed that the F-actin ring-like structure encompasses the cytoplasmic materials and acts as an efficient membrane-free compartment (Fig. 2c). On the contrary, when F-actin flow was disrupted by perturbing filament polymerization and by inhibiting myosin-II activity, nanoparticles failed to accumulate and were found homogeneously distributed throughout the cytoplasmic space of the droplets (Supplementary Figure 6). To characterize the physical properties of the confined cytoplasmic extracts we monitored the diffusion behavior of passive tracers. We tracked the motion of fluorescent latex beads (300 nm diameter) and extracted their mean square displacements for three conditions of confined egg extracts (Fig. 2e and Methods): meiotic extracts in absence of F-actin growth, egg extracts supporting F-actin growth prior to ring-like formation, and egg extracts after the completion of the ring-like formation (see steric trapping occurs within 5 minutes (Fig. 2b and Movie 2). Beads were freely diffusing within the extract cytoplasm for each condition except for the beads that were trapped within the F-actin ring structures. In this latter case beads were completely immobile reflecting the very dense nature of the cytosolic phase confined within the F-actin ring organization. We next estimated the corresponding diffusion coefficients from the bead mean square displacement and found a value of 0.14 μm^2^.s^−1^ for extracts incompetent for F-actin growth, consistent with previous measurements^63^, and 0.07 and 0.05 μm^2^.s^−1^ for egg extracts prior to and after ring-like formation, respectively (Fig. 2e and Methods). These results suggest that the physical property of the cytoplasm external to the confined cytoplasmic space behaves the same irrespective of the presence or absence of the F-actin ring, whereas the cytoplasm confined within the actin-ring behaves like a solid phase for micrometric assemblies.

Altogether our observations within confined egg extracts show that the dynamic state of F-actin meshwork defines two different spatial distributions of nanoparticles and cytoplasmic materials. The nanoparticles were either concentrated by the F-actin meshwork or homogeneously distributed throughout the cytoplasm in cases where F-actin flow was impeded. This membrane-free confinement powered by F-actin flow provides a localized concentration enhancement of nanoparticles, and serves as an active mechanism for the generation and the maintenance of a cytoplasmic partitioning.

### Signaling switch mediated by F-actin flow and active confinement to trigger microtubule aster assembly

**Figure 3:**
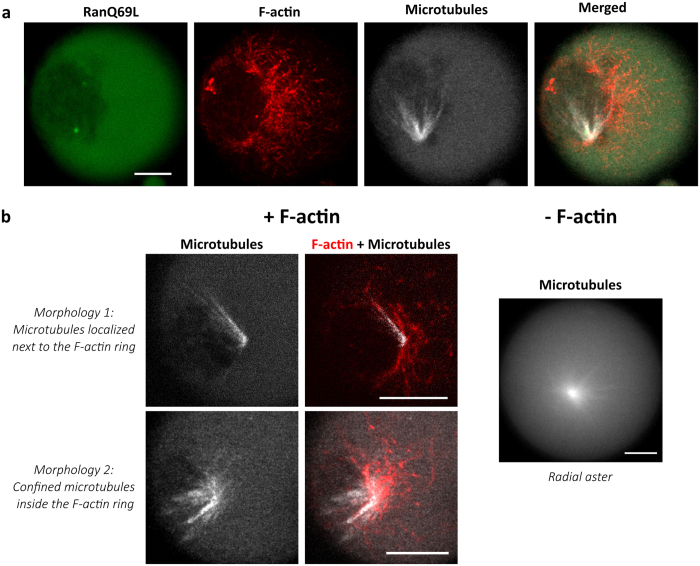
Characterization of asters nucleated with RanQ69L in an F-actin intact cell extract. (**a**) Microtubule asters nucleated with 8 μM of RanQ69L. The microtubules are localized and confined within F-actin ring. mCherry-labeled RanQ69L observation indicates an homogenous distribution of Ran within the droplet. (**b**) Two types of morphologies are observed when microtubule nucleation is triggered in presence of F-actin: asymmetric asters localized inside the F-actin ring and asymmetric asters localized outside the F-actin ring. In absence of F-actin we observed a radial aster. F-actin was labeled with Utr-GFP and fluorescent (TRITC) tubulin was used to visualize microtubules. Scale bars are 10 μm.

To evaluate if the mechanism of cytoplasmic confinement could spatiotemporally direct the activation of biochemical signaling pathways, we have designed a proof-of-concept experiment using small GTPase proteins that exhibit ultrasensitivity, *e.g.* a strong nonlinear input-output relationship (Fig. 1c). We focused our study on the RanGTP pathway and more precisely on RanQ69L, a mutant of Ran locked in the GTP state which promotes the nucleation of microtubule structures into radial asters in *Xenopus* egg extracts^58^. In order to verify that signaling triggered by RanQ69L has the same activation profile in egg extracts supporting F-actin assembly, we monitored the efficiency of aster formation as a function of RanQ69L concentration (0 to 8 μM) in both unconfined and confined extracts. In bulk conditions (unconfined extracts), aster formation exhibits a sigmoid dependence on RanQ69L concentration with a concentration threshold around 4 μM both in presence and absence of F-actin flow (Supplementary Figure 7). Experiments performed with confined extracts display a sharper sigmoidal shape than unconfined extracts, but the threshold for polymerization is about 3 to 5 μM for all conditions (Supplementary Figure 7e). Confocal microscopy revealed that asters nucleated with RanQ69L above the nucleation threshold have their pole positioned next to the F-actin ring structure (Fig. 3a, Supplementary Figure 8a). Interestingly, this localization of microtubule based-structures is observed despite the homogeneous spatial distribution of mCherry-labeled RanQ69L within the droplet (Fig. 3a, Method). We were able to detect two types of morphologies in this condition: asymmetric asters localized inside the F-actin ring and asymmetric asters localized outside the F-actin ring (Fig. 3b left). This is in contrast with radial asters observed in extracts incompetents for actin (Fig. 3b right). In addition, both the morphology and the localization of the asters next to the F-actin ring were similar regardless of the concentration of Ran used (4–10 μM) (Supplementary Figure 8b).

**Figure 4:**
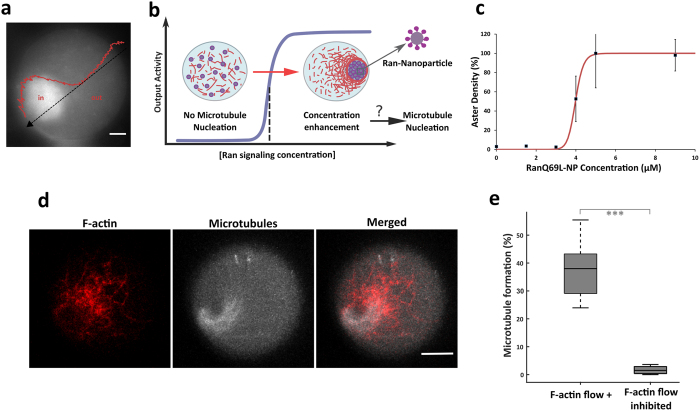
Signaling switch triggered by F-actin flow and by the active confinement of Ran-nanoparticles. (**a**) Estimation of the concentration enhancement driven by F-actin self-organization by quantifying the lysotracker spatial distribution along the droplet diameter. (**b)** Schematic of the proof-of-concept experiment. GTPase RanQ69L are grafted on nanoparticles and the complexes are dispersed within cell extract droplets at a concentration level below the threshold required for microtubule growth. The F-actin flow conveys and confines the Ran-nanoparticles within the F-actin ring-structure to eventually induce a concentration enhancement of Ran in a restricted area, which may lead to activate microtubule nucleation.(**c)** Ran-nanoparticles control microtubule assembly with an activity characterized by a sigmoidal concentration dependency. (**d**) Confocal observation of a microtubule-aster formation induced by the active confinement of Ran-NPs (initially added at a concentration under nucleation threshold, 500 nM). Microtubules are stained with rhodamine-labeled tubulin, and F-actin with Utr-GFP. (**e**) Quantification of the efficiency of the signaling switch: percentage of droplets containing an aster in presence or absence of F-actin flow. The box plot shows the median (central mark), the 25^th^ and 75^th^ percentiles (edges of the box); the whiskers extend to the most extreme data points that are not considered as outliers.

Microscopic observations of the concentration increase of acidic organelles during F-actin contraction (2–5 fold) suggest a hypothetical mechanism of concentration enhancement of mesoscopic objects (Fig. 4a). To implement a biochemical switch mediated by F-actin flow, we used 120 nm nanoparticles as platforms to locally concentrate RanQ69L (Fig. 4b). We first conjugated RanQ69L to nanoparticles (Ran-NPs) and verified the functional activity of the proteins grafted at the nanoparticle surface (Fig. 4c, Methods). The formation of microtubule asters triggered by Ran-NPs exhibited a similar sigmoidal dependence on concentration and nucleation threshold, indicating that conjugating RanQ69L to nanoparticles did not perturb aster formation^56^. Next, we examined how local concentration increase of Ran-NPs mediated by F-actin flow impacts microtubule nucleation. Initially, the Ran-NPs were dispersed homogeneously throughout the droplet cytoplasm at a concentration below the microtubule nucleation threshold (between 0.5 and 1.5 μM). Within five minutes, the nanoparticles were transported by the dynamics of F-actin leading to a strong local enrichment of Ran-NPs (Fig. 2b and steric trapping occurs within 5 minutes (Fig. 2b and Movie 1). Remarkably, after 40 minutes we observed the formation of microtubule-based structures (Fig. 4d and Supplementary Figure 9). Confocal observations showed that the microtubules assembled into aster-like structures or polarized arrays localized either within the F-actin contractile ring or in the vicinity of the microfilament structure (Fig. 4d and Supplementary Figure 9). In some cases we observed the bending of microtubule fibers suggesting that F-actin contractility induces a confinement sufficient to constrain microtubule growth. From these observations we estimate that the forces applied on the microtubule fibers by the F-actin are on the order of 10 *pN* (see Methods). To assess the robustness and efficiency of the signaling switch, we performed systematic studies showing that microtubule fibers were found in more than 35% of the total number of observed contractile droplets (six independent experiments, n = 391 droplets; Fig. 4e), whereas almost no microtubule fibers were detectable in confined extracts when F-actin flow was impeded with cytochalasin-D and blebbistatin (six independent experiments, n = 458 droplets; Fig. 4e). This indicates that the accumulation of Ran-NPs was sufficiently efficient to trigger the nucleation of microtubules, therefore surpassing the local Ran concentration threshold. In addition, the cytoplasmic accumulation of unconjugated nanoparticles within extracts failed to trigger aster assembly, ruling out the possibility that the F-actin flow concentrated non-specific microtubule nucleation factors associated with the nanoparticles. Moreover the absence of bright clusters when monitoring fluorescent proteins suggests that protein aggregation, which could serve as a non-specific protein scaffold, is not occurring inside the F-actin ring (Fig. 3a, Supplementary Figure 3). Thus, the active confinement of Ran-NPs by F-actin self-organization induces a robust signaling switch that triggers microtubule assembly.

### Signaling switch mediated by F-actin-based polymeric scaffolds

**Figure 5:**
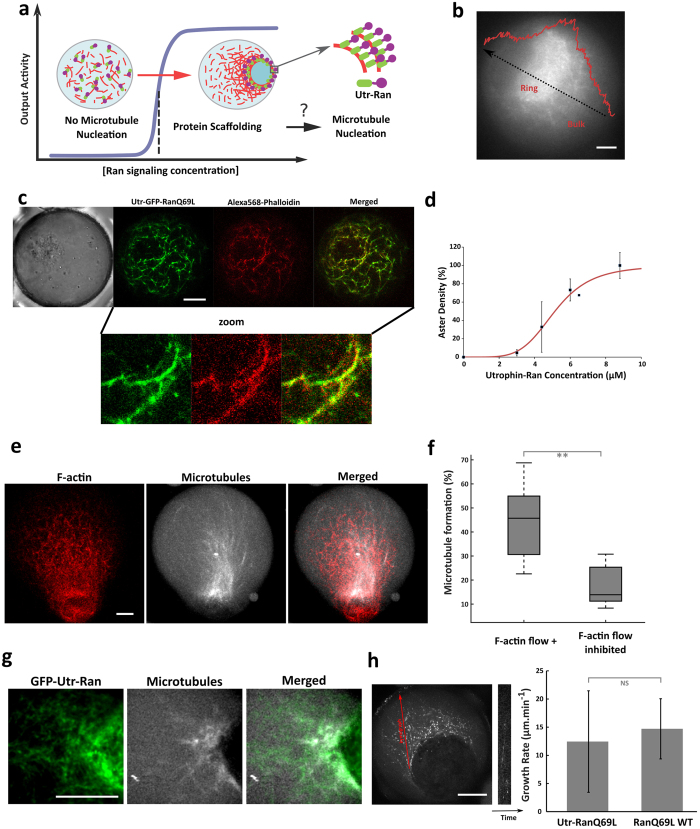
Signaling switch mediated by F-actin polymeric scaffolds. (**a**) Principle of the proof-of-concept experiment examining how F-actin could act as polymeric scaffold hubs to recruit regulatory proteins. RanQ69L is fused to the CH domain of utrophin to target F-actin filaments. Contraction and F-actin density increase may drive the concentration increase of Utr-Ran in a restricted area to trigger microtubule polymerization. (**b**) Evaluation of the fold increase in density of F-actin meshwork after contraction. (**c**) Co-localization of Utrophin-emGFP-RanQ69L (green) with F-actin meshwork (Alexa-568 Phalloidin, red). (**d**) Utr-RanQ69L induces microtubule assembly in confined extracts with a sigmoidal concentration dependency. (**e**) Confocal observation of microtubule formation induced by Utr-RanQ69L under nucleation threshold (500 nM) in confined extract. Microtubules are stained with rhodamine-labeled tubulin, and F-actin with Utr-GFP. **(f**) Quantification of the efficiency of the signaling switch mediated by F-actin flow: number of droplets containing microtubule arrays with F-actin flow and with the inhibition of F-actin flow. Observations were done with confocal microscopy. (**g**) Confocal observation of microtubules induced by GFP-Utr-Ran under nucleation threshold (500 nM). Microtubules are stained with rhodamine-labeled tubulin. (**h**) Left: typical kymograph extracted from steric trapping occurs within 5 minutes (Fig. 2b and Movie 3. Microtubules are labeled with EB1-GFP. Right: quantitative analysis of the growth rate extracted from the kymographs. Growth rates were measured for microtubules assembled using Utr-RanQ69L that are recruited to F-actin scaffolds (initial concentration at 500 nM) and using RanQ69L (6 μM). Mean and standard deviation are plotted. The measured growth rates are similar between these two pathways for growth (Utr-RanQ69L and RanQ69L). Scale bars are 10 μm.

Given the high-order organization and the large density of F-actin meshwork that is assembled during contraction, we next investigated if the direct recruitment of Ran along the microfilaments could trigger sufficient downstream signaling activities to nucleate microtubules (Fig. 5a). Microscopic observations suggest that the density in polymeric actin can reach a 3–8 fold increase during contraction (Fig. 5b), suggesting a hypothetical mechanism of concentration enhancement given that F-actin could act as polymeric scaffold to recruit proteins. To evaluate this concept, we engineered RanQ69L to specifically target F-actin filaments (Fig. 5a) by fusing RanQ69L to the calponin homology domain of utrophin (Utr-RanQ69L). We first verified the capacity of the utrophin domain to bind filaments by co-localizing GFP-Utr-RanQ69L with fluorescent actin filaments (labeled using Alexa-568 Phalloidin, Fig. 5c). We also studied the perturbation of Utr-Ran (500 nM) on the F-actin meshwork structure and dynamics (concentration used in the signaling switch experiments). We found that the ratio between the droplet diameter and the ring diameter was identical in presence or absence of 500 nM of Utr-Ran (Supplementary Figure 2). The size of the F-actin meshwork was also evaluated and found to be slightly increased from 1.1 μm to 1.2 μm in the presence of Utr-Ran supplemented in the extract (Supplementary Figure 3a). On the Supplementary Figure 3b, one can see that the mean F-actin velocity (94 nm.s^−1^) in absence of Utr-Ran is slightly smaller in presence of 500 nM of Utr-Ran (72 nm.s^−1^). Second, we tested the capacity of the chimera protein Utr-RanQ69L to induce microtubule nucleation. Achieving a homogenous distribution of Utr-RanQ69L in the cytoplasm, by impeding F-actin filament formation and consequently any scaffolding effect of RanQ69L, was a prerequisite for accurately assessing the functionality of Utr-Ran in absence of scaffolding. We then quantified the aster density as a function of Utr-RanQ69L concentration and again found a sigmoidal concentration dependence, with a nucleation threshold of 5 μM (Fig. 5d). The timescale for aster formation was similar between RanQ69L and Utr-RanQ69L (between 20 and 30 minutes) indicating that Utr-RanQ69L remains functional. Finally, to evaluate F-actin scaffolding can mediate a signaling switch, we added Utr-RanQ69L to the egg extract at an initial concentration under the nucleation concentration threshold (500 nM). Remarkably, the assembly of the F-actin meshwork triggered the nucleation of microtubule filaments in less than one hour (Fig. 5e and Supplementary Figure 10a). We quantified this effect and found that microtubule assemblies occurred within 43% of the droplets (7 independent experiments, n = 246 droplets), whereas only 17% of the droplets contained microtubules when the F-actin flow was disrupted (7 independent experiments, n = 259 droplets) (Fig. 5f). Therefore, almost no microtubule nucleation was observed when F-actin flow was disrupted, showing the specificity of the actin scaffold. This suggests that non-specific protein aggregation is not involved in the microtubule nucleation process. The few microtubule nucleation events observed in absence of F-actin flow could originate from remaining micrometric F-actin patches (despite drug treatment) that could scaffold Utr-RanQ69L (Supplementary Figure 11).

Microtubule filaments appeared to be supported by the actin meshwork. We also generally observed that microtubules assembled into polymeric arrays rather than into asters. These structures were found aligned with F-actin fibers (Supplementary Figure 10b) and co-localized with GFP-labeled Ran-Utr (Fig. 5g). Further characterizations of the microtubule-based structures were performed using EB1-GFP to visualize microtubule plus-end dynamics. A typical example of microtubule dynamics is shown in steric trapping occurs within 5 minutes (Fig. 2b and Movie 3, which suggests that the microtubule arrays mediated by Utr-RanQ69L are polarized with a plus-end dynamic having a growth rate between 5 and 20 μm.min^−1^; this range of velocity is similar to that obtained with RanQ69L wild type (Fig. 5h).

Altogether we have demonstrated how the artificial scaffolding mediated by F-actin could drive the accumulation of Ran with an enhanced concentration sufficient to trigger the activation of microtubule nucleation, forming an asymmetric polarized array of fibers.

Interestingly, the microtubules organized into two different patterns according to the mode of localization of Ran by F-actin: confinement of Ran-NPs within F-actin ring and scaffolding along actin polymers (Supplementary Figure 12). In the case of Ran-NPs microtubules formed a single asymmetric aster, whereas in the case of Utr-Ran microtubules organized into a polarized array of fibers without pole organization (Fig. 5e). This suggests a mode of spatial organization of microtubules that depend on F-actin state and on the localization of nucleation.

## Discussion

F-actin structures and dynamics drive numerous essential functions when they are assembled at the cortex of cells as well as within the cytoplasm. We have shown that the combination of *in vitro* reconstitution of a confined cytoplasm with synthetic strategies imparted two novel functions to F-actin dynamics: first, the membrane-free confinement of signaling proteins, and second, the scaffolding of signaling proteins along actin polymers. In this context, F-actin self-organization into contractile meshwork drives the triggering of the RanGTP pathway, an archetypal of ultrasensitive signaling switch.

From the perspective of synthetic biology, our *in vitro* approach allowed us to explore and implement artificial and novel functions by engineering the F-actin spatial organization and signaling processes^64^,^65^,^66^. Our bottom-up approach is generic, versatile and modular, and could be extended to other signaling proteins, such as kinases, that exhibit non-trivial dynamics through collective effects.

One important difference between our *in vitro* system and living systems concerns the regulation of actin cytoskeleton, which occurs in cells in a reversible manner, thus providing a richer dynamic of control. An improvement of our assay from droplets of egg extracts to vesicles of egg extracts made of a lipid bilayer may allow the use of drugs to assess more precisely the dynamic properties of such signaling switches induced by F-actin self-organization. F-actin dependent-formation of microdomains in dendritic spines and during T cell signaling are some of the many possible roles for microfilament control of signaling pathways in living cells^17^,^19^. Our *in vitro* assay isolates simple design principles underlying the control of signaling pathways by cytoplasmic F-actin self-organization to tune signaling processes: either by docking signaling proteins to a mesoscopic-like structure sufficiently large to be non-specifically confined by a contractile meshwork, or the specific recruitment of signaling proteins along actin microfilaments, which serve as linear and rigid scaffolding polymers. One perspective will be the implementation of the strategy into living cells in order to artificially couple F-actin spatiotemporal organization and signaling events. This may help to examine if the widespread occurrence of F-actin structures throughout living cells could be used to shape signaling events.

Furthermore, there are mounting interests in studying how phase transitions generate membrane-free micro-domains in the cytoplasm that eventually regulate functions or signaling pathways^8^,^9^,^12^,^13^. These mechanisms generally operate near thermodynamic equilibrium, and interestingly we found that out-of-equilibrium structures powered by F-actin organization can contribute in partitioning the cytoplasm and in shaping signaling processes in space and time.

Finally our minimal system could be extended to examine possible mechanisms of crosstalk between actin and microtubules, which could be reconstituted in a controlled manner.

## Material and Methods

### Expression and purification of recombinant proteins

The plasmids for *E. coli* expression of RanQ69L (pQE32-Ran, 6His Tag) and EB1-GFP were kindly provided by Iain Mattaj (EMBL) and Ron Vale (UCSF) respectively. The plasmids Utr-Ran, Utr-GFP, and GFP-Utr-Ran for *E. coli* expression were cloned from pSPE3 GFP Utrophine plasmid^21^ in a pET28. Expression of plasmids and purification of recombinant proteins (RanQ69L, EB1-GFP, Utr-Ran, Utr-GFP, Utr-GFP-Ran) were realized using standard protocols.

### Reagents

ATP, DTT, creatine phosphate, cytochalasin D, latrunculin-A, blebbistatin, creatine phosphokinase and mineral oil (M5904) were purchased from Sigma-Aldrich (St Louis, MO). Poly(12-hydroxystearic acid) (PHS) and poly(ethylene oxide) (PEO-30) are commercially available (Arlacel P135) and were purchased from UNIQEMA. Tubulins, labeled with Rhodamin or with FITC, were ordered from Cytoskeleton Inc. (Denver, CO). Membrane tracker, lysotracker and Alexa-fluor 568 Phalloidin were purchased from Life Technologies. 120 nm and 300 nm nanoparticles were purchased from Ademtech. 50 nm nanoparticles were purchased from Micromod.

Cytostatic-factor-arrested (CSF) *Xenopus laevis* egg extracts, which correspond to active cytoplasm of oocytes arrested in metaphase II of meiosis, were prepared as previously described^67^, with the following modifications: no cytochalasin D was added and all steps were carried at 4 °C^41^,^68^. All reagents for buffer preparation were purchased from Sigma-Aldrich.

### Microtubule and actin assembly

Microtubule structures were assembled using metaphase *Xenopus laevis* egg extracts, containing an ATP regenerating system (1 mM ATP, 10 mM creatine phosphate, 100 μg/μL creatine phosphokinase, final concentrations), incubated in the presence of RanQ69L, Ran-NP complexes, or UtrCH-RanQ69L, at the final concentration indicated in the manuscript for 30 min at 19 °C. Microtubules were labeled either with Rhodamin-labeled tubulin at 100 nM final (Cytoskeleton Inc.), Fluorescein-labeled tubulin at 100 nM final (Cytoskeleton Inc.), or EB1-GFP at 150 nM.

Actin structures were assembled using metaphase *Xenopus laevis* egg extracts, containing an ATP regenerating system (1 mM ATP, 10 mM creatine phosphate, 100 μg/μL creatine phosphokinase, final concentrations). The actin flow was disturbed using blebbistatin at 130 nM final and cytochalasin D at 2 μg/mL final or latrunculin at 25 μM final. Actin was labeled with GFP-utrophin at 110 nM final or Alexa-Fluor 568 Phalloidin at 50 nM final.

### Imaging and Data Analysis

Fluorescence imaging of microtubule asters and actin network was performed using an IX81 (Olympus) and X60 (PlanApo, NA 1,42) oil objective, equipped with an EM-CCD camera (electron multiplying CCD, C9100-02, Hamamatsu, Corporation), and a LED system of illumination (Spectra X, Lumencor). Microscope settings and functions were controlled using Simple PCI software (Hamamatsu). Image analysis was performed using ImageJ Software, Matlab, and Simple PCI software. Confocal microscopy was performed with a Zeiss LSM 710 META laser scanning confocal microscope using X63 (PlanApochromatic, NA 1,4) objective. Image analysis was performed using LSM Software Zen 2009 and ImageJ.

To quantify the concentration enhancement of organelles within the F-actin ring-like structures, contractile cell extracts were supplemented with lysotracker (Lysotracker Green DND-26 final concentration 2 μM, Fig. 4a). We first monitored F-actin droplets and computed the intensity profile to quantify the lysotracker spatial distribution along the droplet axis for both contractile and non-contractile F-actin states. After normalization and averaging, we obtained fluorescent profiles that were deconvoluted to correct the volume contribution. Then the concentration enhancement was estimated by computing the ratio between the maximum and the minimum of the intensity profile. The same procedure was applied for computing the concentration enhancement of labeled F-actin at late F-actin contraction (Fig. 5b).

To estimate viscosity of cell extracts in different conditions, we monitored the Brownian motion of latex beads (328 nm diameter, Estapor). The trajectories of the beads were then recovered thanks to IcY software^69^. Mean Square Displacement (MSD) analysis was computed using Matlab and the diffusion coefficient of the beads was obtained from the 25 first percent of the MSD slope. For the experiments performed after ring formation, the motions of immobile particles within the F-actin ring have not been considered for the estimation of the viscosity.

We estimated the meshsize of the network from confocal images. We plotted intensity profiles and then measured the distance between two fibers. We only took the values that were above the diffraction limit of our optical system (500 nm).

Student’s t-tests were performed with Matlab. For the interpretation of the p-values: NS means there is no significant difference between the two distributions. One star means pvalue <0,05, two stars means pvalue <0,01, three stars means pvalue <0,001.

To quantitatively assess the degree of colocalization between the actin and microtubule structures we used the ImageJ plugin “Coloc 2” to plot the 2d-histograms and get the Pearson’s coefficients for the two representative images in Supplementary Figure 10b.

### Visualization of Ran using mCherry reporters

In order to see how RanQ69L is distributed within the droplet, we generated a dimer between Ran and mCherry using FRB and FKBP dimerizing system. Briefly, we formed dimers with FKBP-Ran and FRB-mCherry in a premix during 5 minutes at room temperature ([FKBP-Ran]=112 μM; [FRB-mCherry]=11,2 μM; [Rapamycin]=100 μM). We performed extract experiments with a final concentration of Ran of 8 μM.

### Conjugation of Ran proteins to nanoparticles

Carboxylic acid nanoparticles (iron oxide core, 120 nm diameter) were purchased from Ademtech. The nanoparticles/RanQ69L conjugation was performed as previously described^56^. Conjugation stoichiometry was determined by a semi-quantitative assay using SDS-PAGE electrophoresis.

### Extract-in-Oil Droplet Formations

Cellular extract was encapsulated in droplets via water-in-oil emulsion process. Mineral oil contains a biocompatible block copolymer in order to stabilize emulsion and facilitate observations. This method has been previously described and allows the formation of microtubule asters and active actin meshworks in droplets^40^,^70^. PHS-PEO-PHS block copolymer (Arlacel P135) was first dissolved in mineral oil (0.4 mg.mL^−1^). The *Xenopus laevis* egg extracts containing fluorescently labeled tubulin and/or utrophin-GFP was then added to the block copolymer solution (1% (v CSF/v Oil)) at room temperature. The mixture is gently sheared, by pipetting up and down the solution during few seconds, to generate extract-in-oil droplets. The mechanical dispersion of the biphasic solution formed micrometer-sized extract-in-oil droplets within few seconds. The emulsion is incubated for 20–30 minutes at 19 °C. Then droplets were observed in a time frame comprised between 20 and 60 minutes after mixing of the components. The droplets presenting microtubule formation had a diameter comprised between 10 and 55 μm. Observations were done maximum one hour after the beginning of the incubation. After one hour of incubation, some spontaneous microtubule nucleation could be sometimes observed.

#### Estimation of the forces applied on microtubules

Two mechanisms of force generation could explain the bending of microtubules observed in our observations (Fig. 3d and Fig. 3 – figure supplement 1). First, the forces applied on elongated microtubules could be directly produced by the F-actin meshwork during contraction through both the polymerization of actin microfilaments and the myosin II activity. Second, microtubule fibers during growth can encounter the F-actin ring-like structure that can be considered as a rigid confinement boundary. Consequently when growing microtubules encounter the F-actin ring-like structure, normal compression forces are exerted. These compressive forces eventually cause microtubules to buckle. This buckling instability appears when a critical force, *F*_*c*_, is reached. *F*_*c*_ scales with *N*^*2*^*B/l*^*2*^; *l* is the microtubule length, *N* the number of filaments composing the bundle (the filaments are cross-linked together), and *B* the bending modulus characterizing the stiffness of the microtubule (25 *pN.*μm^2^). *N* is found to vary between 3 to 7 microtubules in egg extracts^70^. Therefore, typical observations indicate that forces of the order of 10 *pN* are applied on microtubules.

## Additional Information

**How to cite this article**: Colin, A. *et al*. Triggering signaling pathways using F-actin self-organization. *Sci. Rep.* **6**, 34657; doi: 10.1038/srep34657 (2016).

## Supplementary Material

Supplementary Information

Supplementary Movie 1

Supplementary Movie 2

Supplementary Movie 3
