# Supplementary material for: Triggering signaling pathways using F-actin self-organization
Source: Sci Rep. 2016 Oct 4;6:34657. doi: 10.1038/srep34657 (PMC5048156; doi:10.1038/srep34657)
Supplement: Supplementary Information [file srep34657-s1.pdf]

Supplementary Information

Triggering signaling pathways using F-actin self-organization

*A. Colin#, L. Bonnemay#, C. Gayrard, J. Gautier, and Z. Gueroui**

Ecole Normale Supérieure, Department of Chemistry

PSL Research University-CNRS-ENS-UPMC

24, rue Lhomond, 75005, Paris, France.

12 Supplementary Figures, 3 Supplementary movies

Correspondence:

Zoher GUEROUI

Phone: +33 1 44 32 24 09

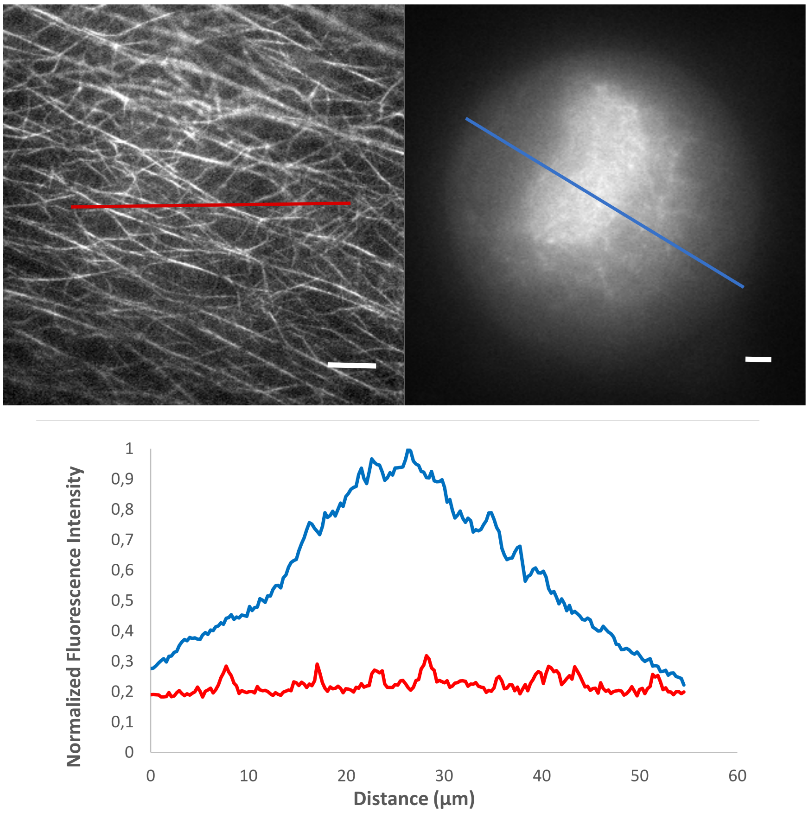

Supplementary Figure 1. F-actin meshwork morphology observed in bulk and in confined droplet. On the left, F-actin meshwork formation polymerized in bulk. On the right, F-actin meshwork formation confined in a droplet. Microfilaments were labeled with Utr-GFP. Intensity profiles quantifying the F-actin spatial density and highlighting the strong enrichment of fibers when F-actin meshwork is contracted. Scale bars are 10 µm.

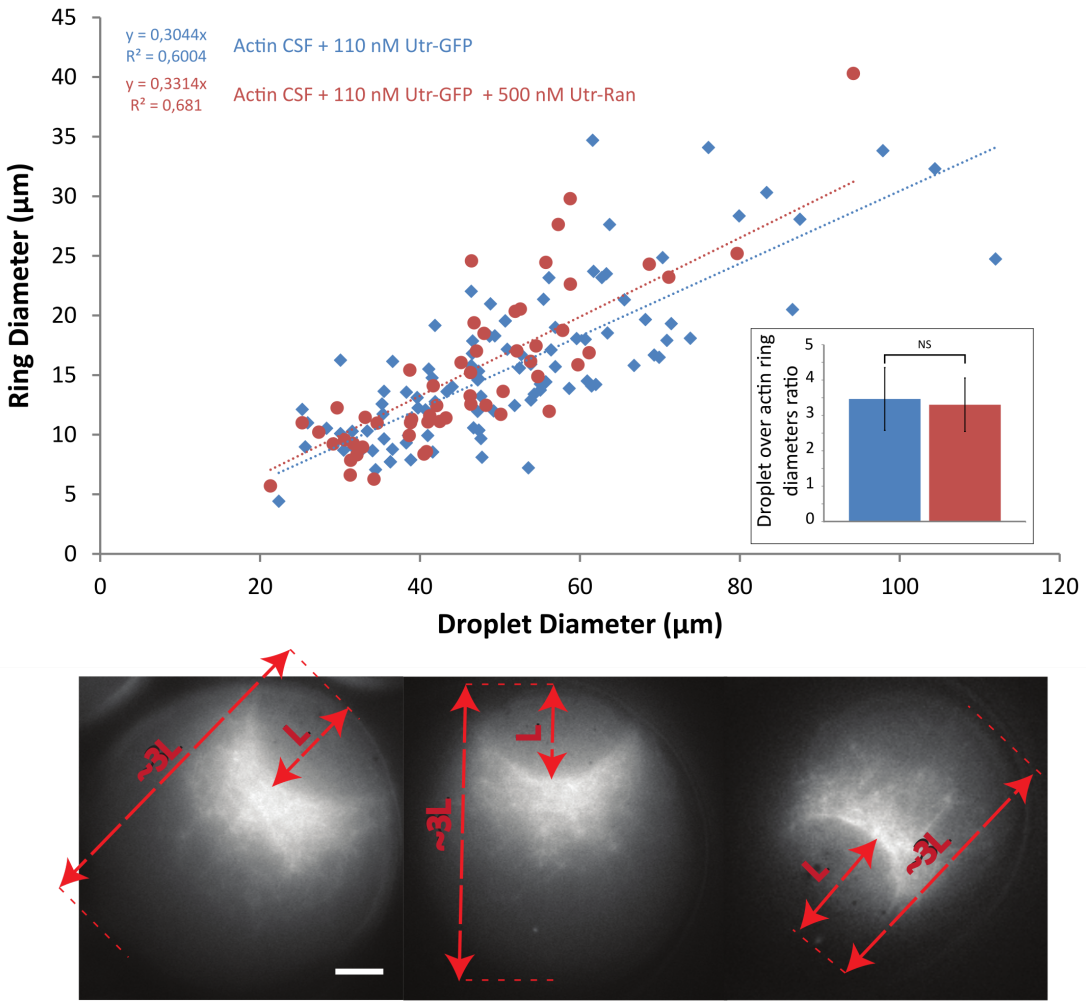

Supplementary Figure 2. The size of the actin ring scales with a ratio of 3 with the size of the droplet. Top: Plot of the F-actin ring structure diameter as a function of the droplet diameter showing a ratio of 3 in ratio between these two diameters. The ratio between the droplet diameter and the ring diameter was identical in presence or absence of 500 nM of Utr-Ran. Bottom part: examples of F-actin ring structures. Scale bar is 10 µm.

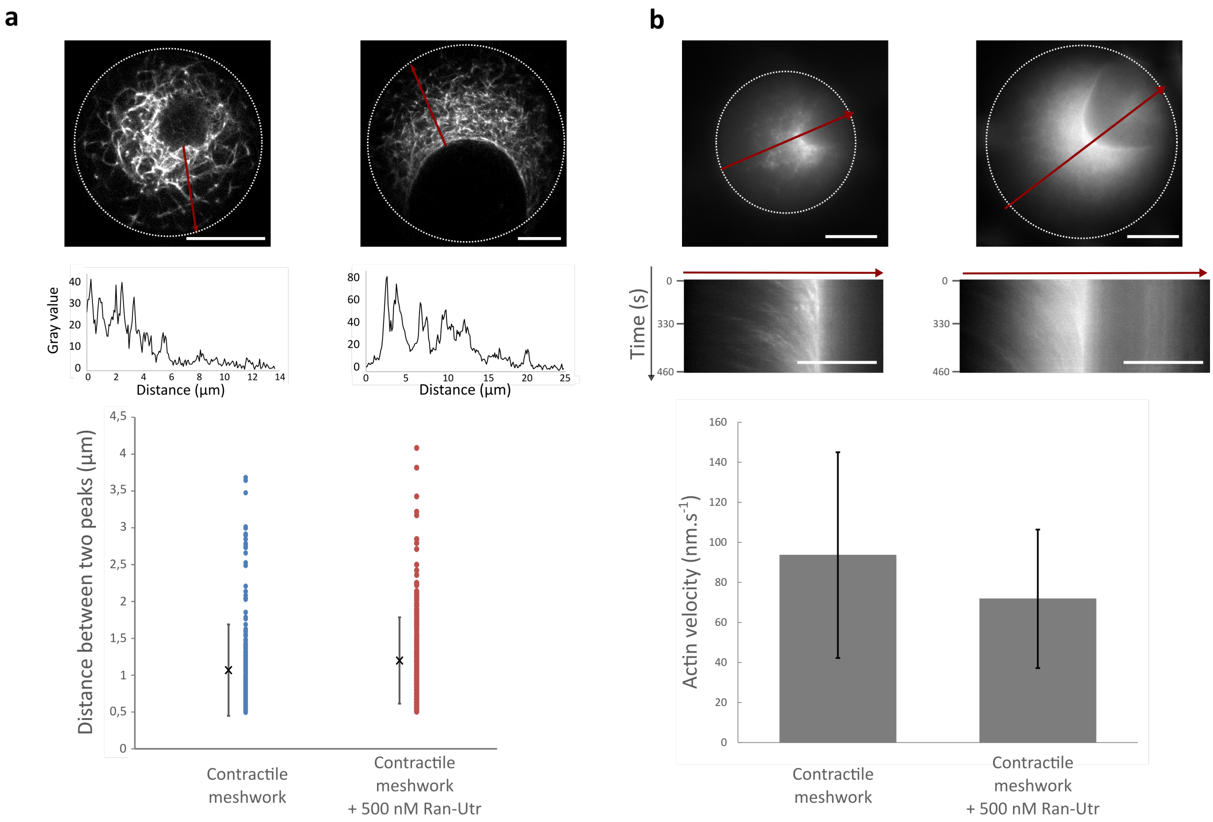

Supplementary Figure 3. Characterization of the F-actin meshwork structure and dynamics. a. Top: Confocal images of F-actin rings obtained in absence (left) or presence (right) of 500 nM Utr-Ran. The plots represent the intensity profiles taken along the red line. To measure the distance between two fibers of the F-actin meshwork, we extracted from the intensity profiles the distances that were larger than the diffraction limit (500 nm). Bottom: Plot of the distribution of distances, mean, and standard deviation (351 and 355 respectively for each case). **b.** Comparison of F-actin flow dynamics in presence or in absence of 500 nM of Ran-Utr. Representative kymographs illustrating the contractile behavior of F-actin. The mean F-actin velocity in absence of Utr-Ran is 94 $nm.s^{-1}$. In presence of 500 nM of Utr-Ran, the mean F-actin velocity is 72 $nm.s^{-1}$. F-actin was labelled with 110 nM of Utr-GFP in both cases. Scale bars are 10 µm.

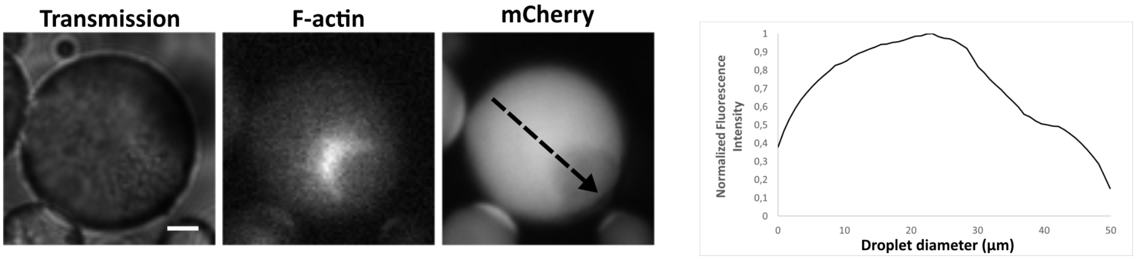

Supplementary Figure 4. The actin ring conveys the cytoplasmic organelle-like materials but not small proteins.

Spatial repartition of cytoplasmic fluorescent proteins within droplets with contractile activity. On the left, we selected examples of droplets containing fluorescent proteins (mCherry). Actin is labeled with utrophin-GFP. On the right, we represent the intensity profile of the fluorescence protein along the droplet diameter (dotted arrow). The distribution of mCherry shows that it is mostly excluded from the ring. Scale bar is 10 μm.

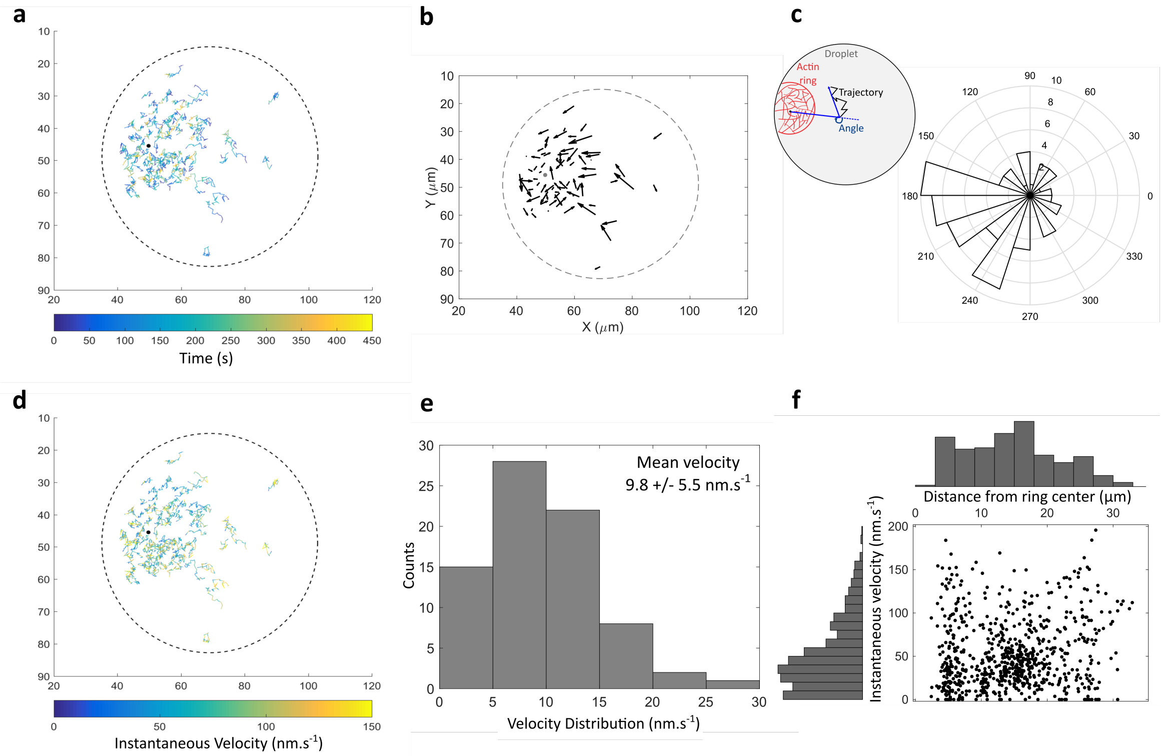

Supplementary Figure 5. Multiple particle tracking to characterize the spatiotemporal dynamics of the nanoparticles during F-actin self-organization.

a. Displacement fields of 300 nm nanoparticles reveal a directed motion toward the F-actin ring. **b.** Mean orientation for each nanoparticle trajectories within the droplet. **c.** Angular distribution of tracked trajectories with respect to the actin ring position. **d.** Velocity field distribution computed for the trajectories of nanoparticles shows that instantaneous velocities are heterogeneous along single tracked trajectories. **e.** Distribution of the mean velocities of each nanoparticle trajectories (mean = 9.8 $nm.s^{-1}$). **f.** The distribution of instantaneous velocity is independent of the nanoparticle localization within the droplets.

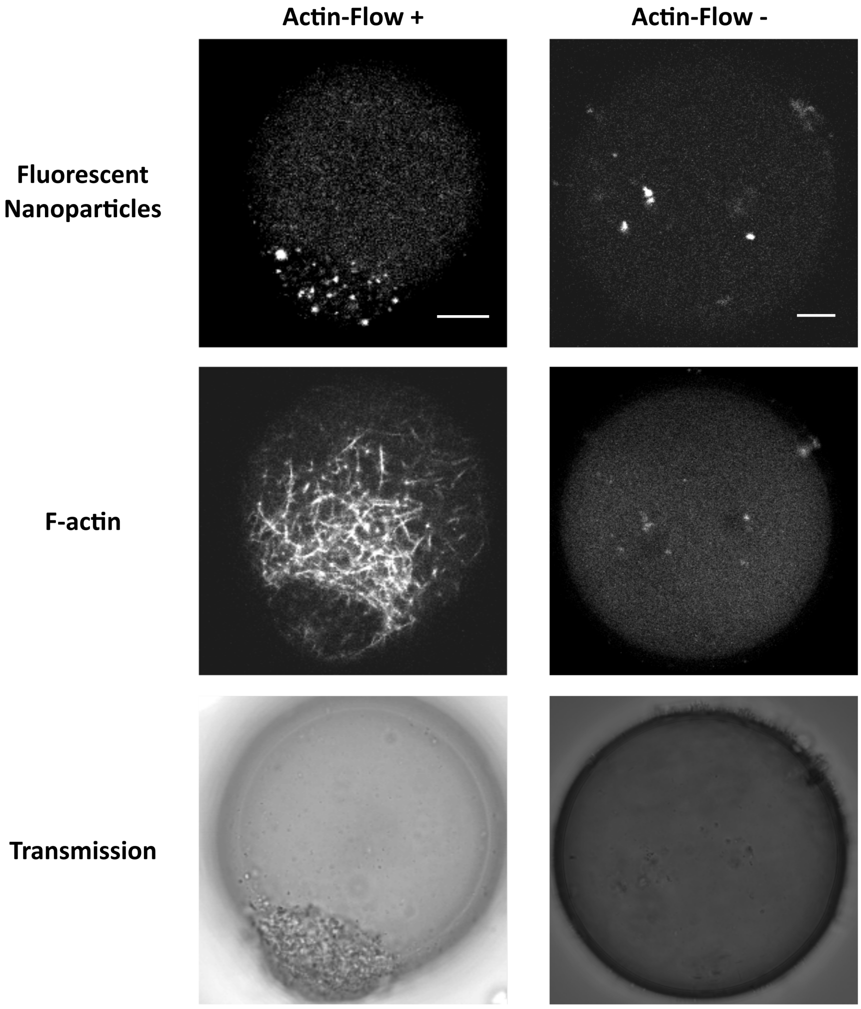

Supplementary Figure 6. The nanoparticles are homogeneously distributed in the droplet in absence of the F-actin flow. Nanoparticles fail in accumulating in a restricted area when F-actin flow is disrupted (using blebbistatin and cytochalasin-D). The nanoparticles are found randomly distributed within the cytoplasm. Scale bars are 10 µm.

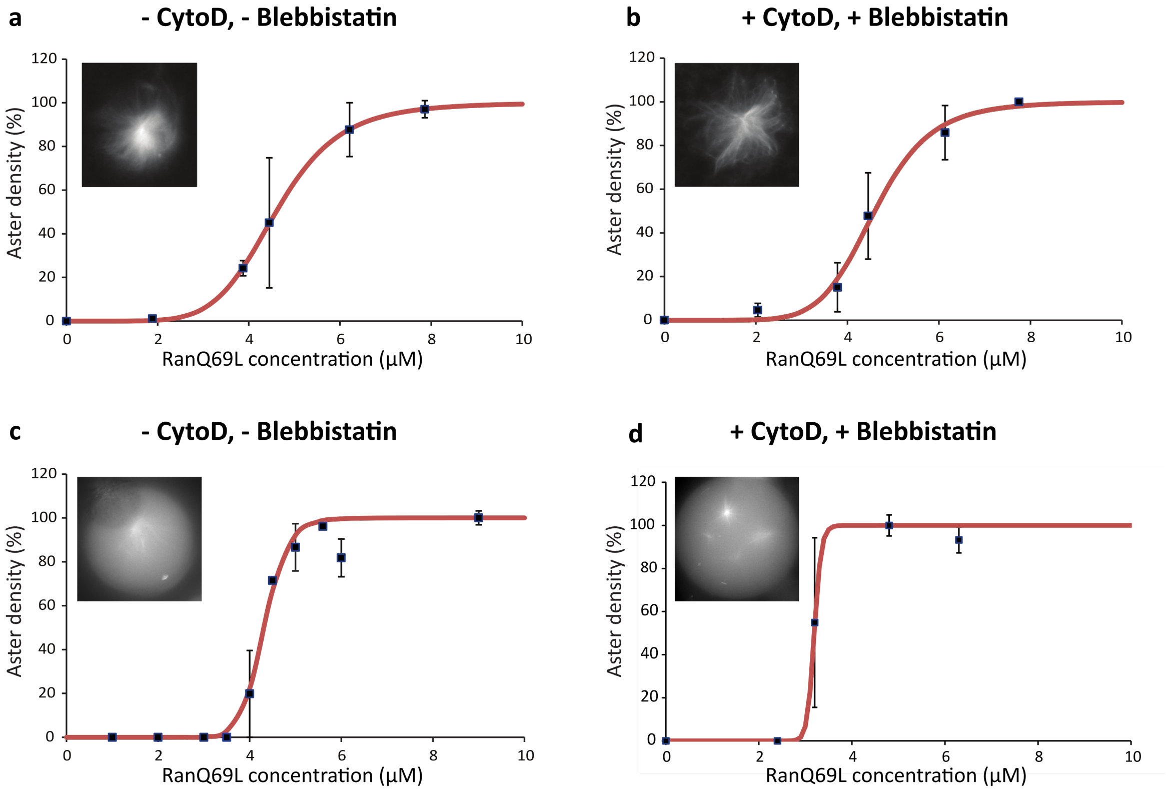

e

Fit-type

$$\frac{\left(\frac{[Ran]}{a}\right)^{b}}{1+\left(\frac{[Ran]}{a}\right)^{b}} \times 100$$

Nucleator	Confinement?	Drugs?	a	b	Figure?
RanQ69L	No	No	4,6	6,6	Supplementary Figure 5a
RanQ69L	No	Yes	4,6	7,5	Supplementary Figure 5b
RanQ69L	Yes	No	4,3	16,6	Supplementary Figure 5c
RanQ69L	Yes	Yes	3,2	42,7	Supplementary Figure 5d
RanQ69L-NPs	No	Yes	4,0	20,2	Figure 4c
Utr-Ran	Yes	Yes	5,1	5	Figure 5d

Supplementary Figure 7. The sigmoidal response of the Ran pathway is conserved in extracts supporting F-actin meshwork formation. Microtubule nucleation is ultrasensitive to the concentration of RanQ69L-GTP in bulk extracts and confined extracts, either in presence or in absence of F-actin contraction. Aster density was quantified by fluorescence microscopy and normalized to reach 100% for the plateau at the highest RanGTP concentration. **a,b**. Aster efficient for experiments performed with unconfined extracts in presence (a) or absence (b) of F-actin contractile activity. **c,d**. Aster efficient for experiments performed with confined extracts in presence (c) or absence (d) of F-actin contractile activity. Thus, the confinement and the F-actin dynamics are not modifying the concentration threshold for aster assembly. **e**. Fitting values of the sigmoidal response of microtubule polymerization as function of RanQ69L concentration. The parameter *a* represents the concentration threshold, whereas *b* represents the slope of the fitted curve (Hill factor).

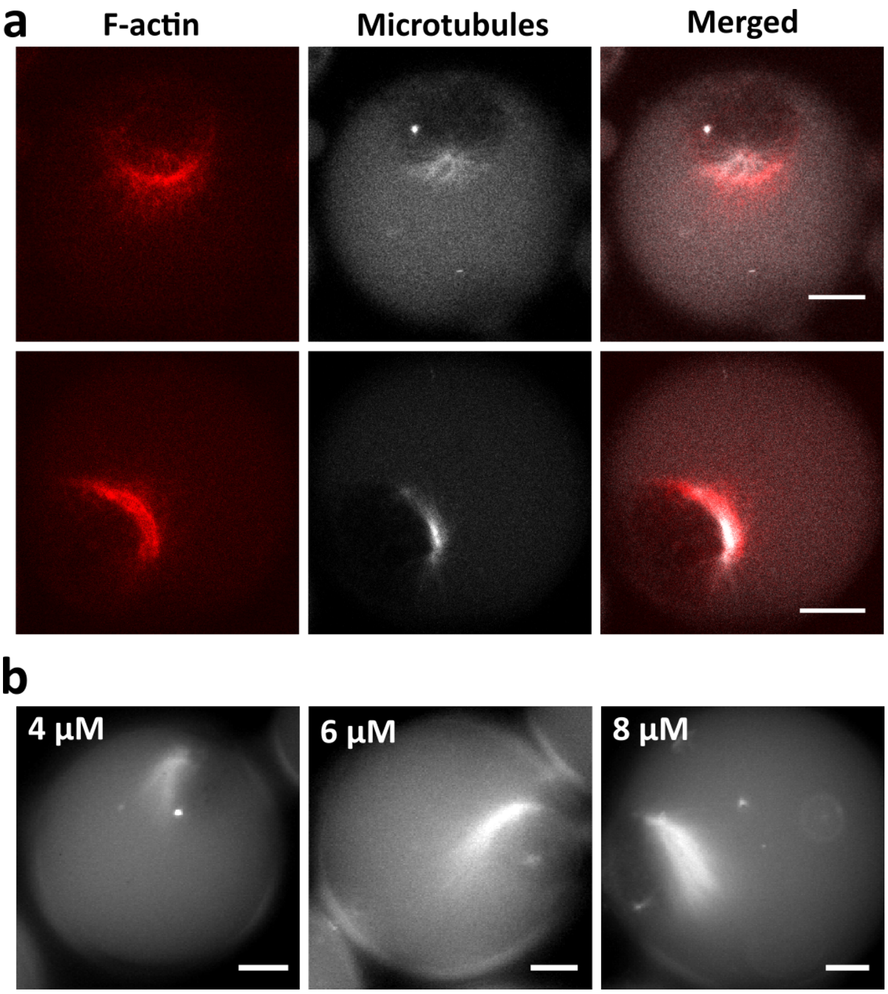

Supplementary Figure 8. Microtubule fibers nucleated with Ran during F-actin meshwork formation are localized next to the microfilaments.

a. Examples of confocal observations showing that microtubule arrays are nucleated next to F-actin ring-like structures. Asters were nucleated from an extract containing 10 µM of RanQ69L. F-actin filaments are labeled with Alexa-Fluor 568 Phalloidin (red channel) and microtubules are labeled with FITC-tubulin (gray channel). **b.** Observations of the morphology of the microtubules nucleated for various concentrations of RanQ69L in an F-actin intact cell extract. Microtubules are labeled with TRITC tubulin. Scale bars are 10 µm.

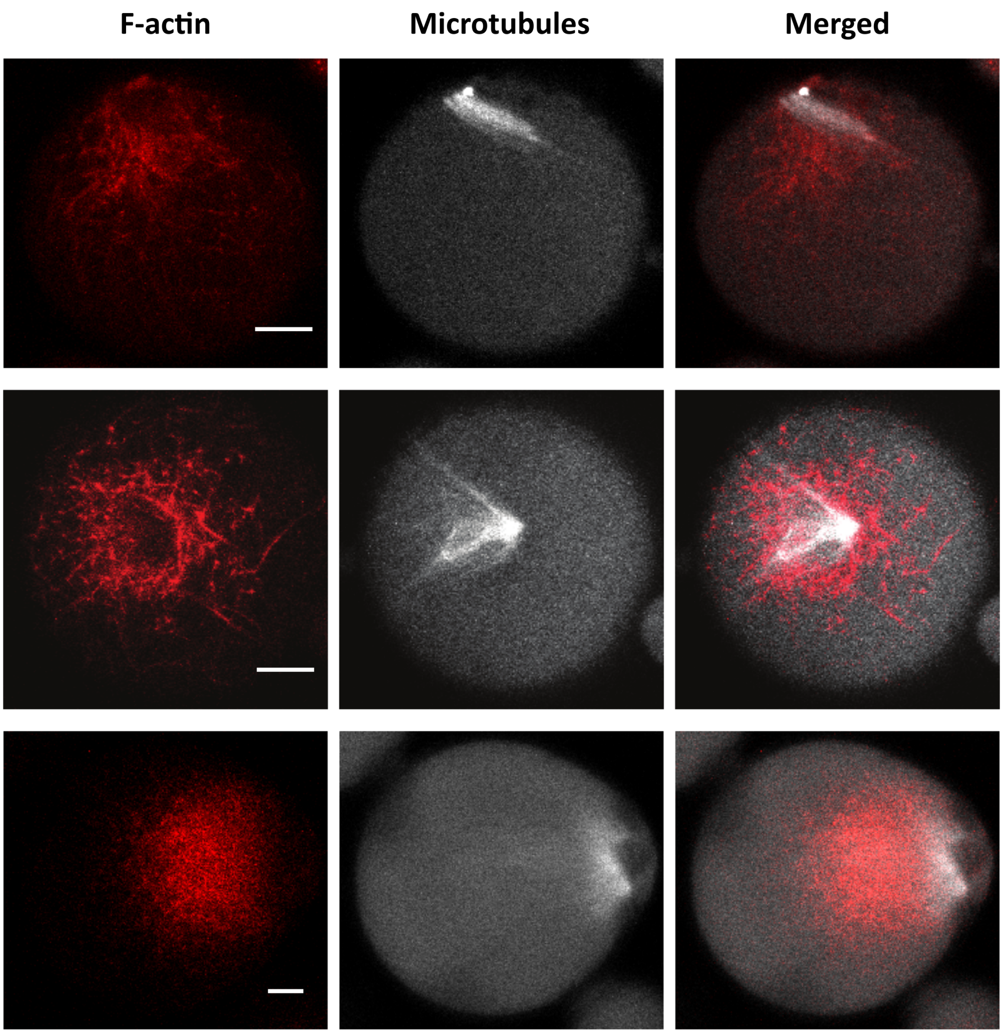

Supplementary Figure 9. Examples of microtubule organizations triggered by F-actin flow and by the active confinement of Ran-nanoparticles

Confocal observations of contractile extract droplets illustrating the colocalization of microtubule arrays with F-actin meshwork. F-actin filaments are represented in red and are labeled with Utr-GFP. Microtubules are represented in gray and are labeled with TRITC-tubulin. Scale bars are 10 µm.

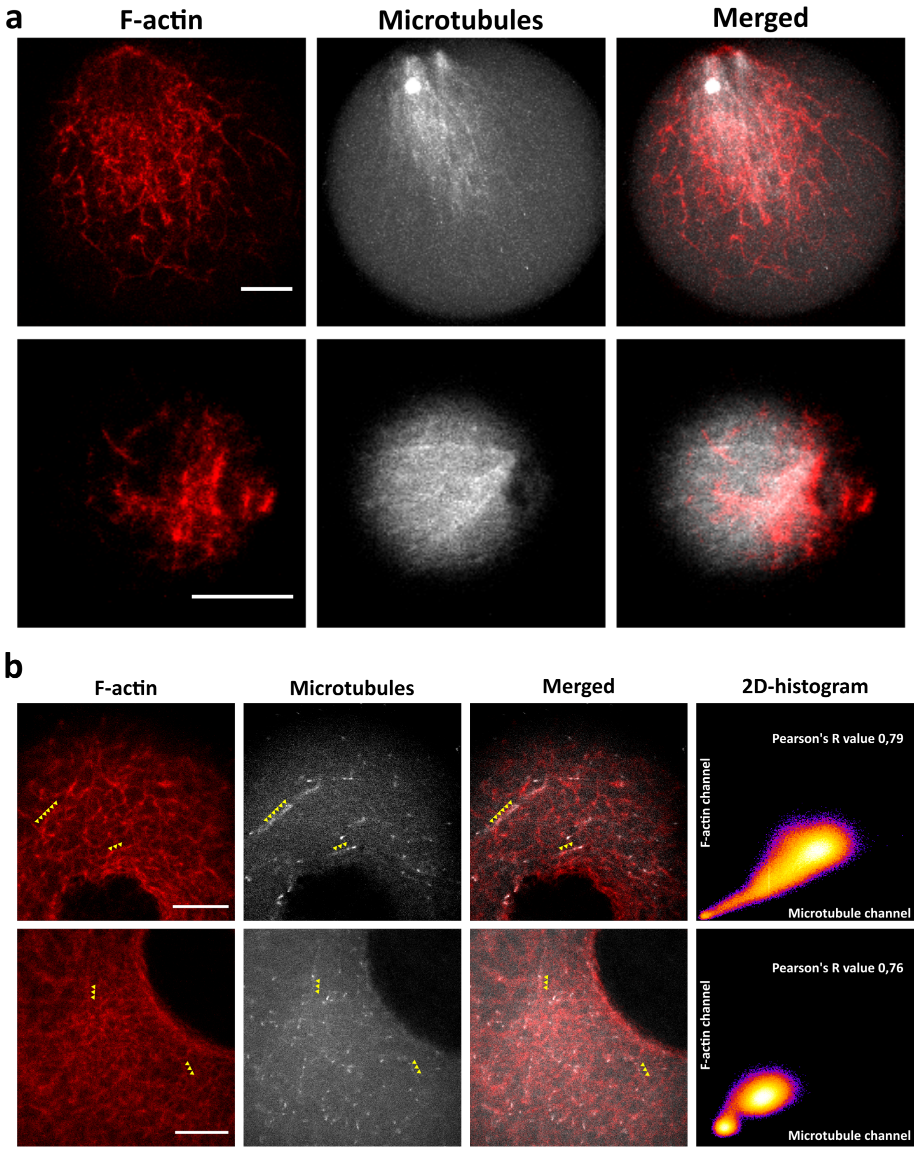

Supplementary Figure 10. Examples of morphologies of microtubule arrays assembled with Utr-Ran scaffolded on F-actin meshwork.

a. Confocal co-localization between microtubule arrays (TRITC-tubulin, gray) and F-actin ring-like structures (Utr-GFP, red). **b.** Left: example of events of co-alignments between microtubules and F-actin fibers (yellow triangles). Microtubules are labelled with EB1-GFP and FITC-tubulin; F-actin is labelled with Utr-dsRed. Scale bars are 10 µm. Right: 2D-histograms representing the quantification of the co-localization between F-actin and microtubule fluorescence channels. Pearson's coefficient is indicating the degree of correlation.

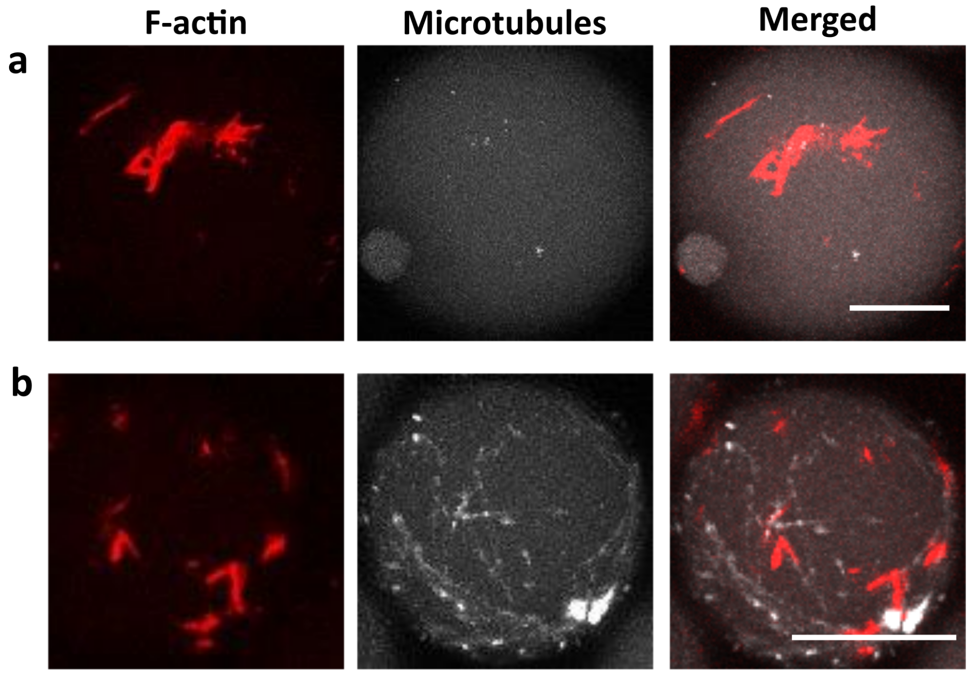

Supplementary Figure 11. Examples of confocal observations of control experiments in confined extracts when F-actin contraction is inhibited. Co-localization of F-actin and microtubules mainly shows an absence of microtubule nucleation within droplets (a) and in some few cases droplets with spontaneous microtubule fibers (b). F-actin is labeled with Alexa-Fluor-568-phalloidin; microtubules are labeled with FITC-tubulin and EB1-GFP. The images presented are Z-projection of confocal images. Scale bars are 10 μm.

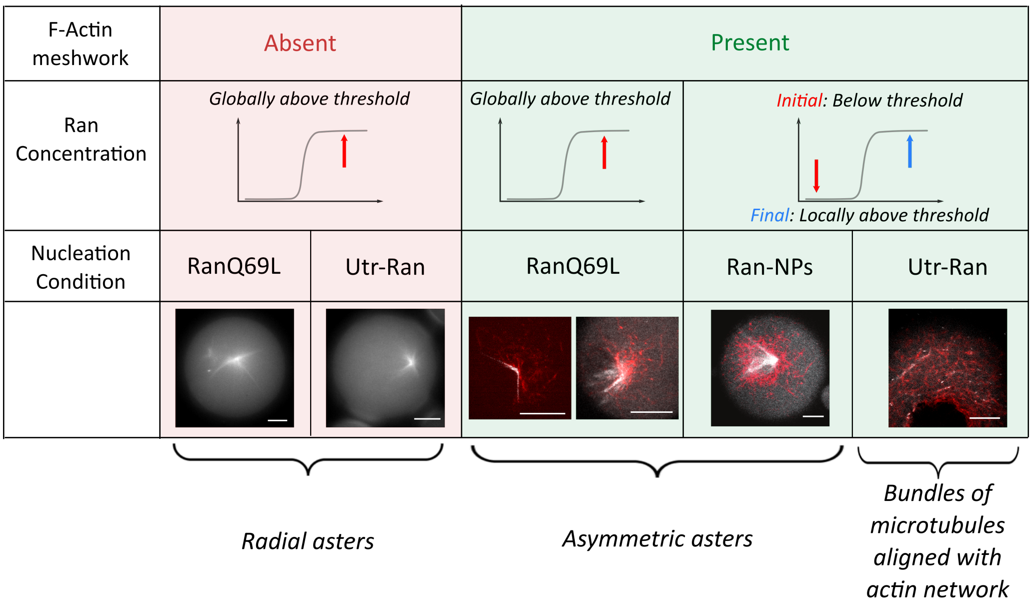

Supplementary Figure 12. Comparison of the microtubule morphologies obtained for different nucleation conditions.

Legends for Supplementary Movies

Supplementary Movie 1

The dynamic accumulation of nanoparticles (300 nm in diameter) during F-actin meshwork contraction occurs in less than 5 minutes. Frames are 10 seconds apart.

Supplementary Movie 2

Examples of movies displaying the motion of fluorescent latex beads (300 nm diameter) that were used for the estimation of the diffusion coefficient of tracers in the cell extract. From left to right: meiotic extracts in absence of F-actin growth, egg extracts supporting F-actin growth prior to ring-like formation, and egg extracts after the completion of the ring-like formation. Movies were recorded with the stream mode of the microscope and frames are 120 ms apart.

Supplementary Movie 3

Time-lapse movie showing the dynamic of microtubule plus-end growth using EB1-GFP Frames are 5 seconds apart.
